# Selection of an Optimal Recombinant Egyptian H9N2 Avian Influenza Vaccine Strain for Poultry with High Antigenicity and Safety

**DOI:** 10.3390/vaccines10020162

**Published:** 2022-01-21

**Authors:** Se-Hee An, Seung-Eun Son, Jin-Ha Song, Seung-Min Hong, Chung-Young Lee, Nak-Hyung Lee, Young-Ju Jeong, Jun-Gu Choi, Youn-Jeong Lee, Hyun-Mi Kang, Kang-Seuk Choi, Hyuk-Joon Kwon

**Affiliations:** 1 Laboratory of Avian Diseases, Department of Farm Animal Medicine, College of Veterinary Medicine and BK21 PLUS for Veterinary Science, Seoul National University, 1, Gwanak-ro, Seoul 88026, Korea; eepdl1201@snu.ac.kr (S.-H.A.); arbre04@snu.ac.kr (S.-E.S.); sjh1243@snu.ac.kr (J.-H.S.); topkin@snu.ac.kr (S.-M.H.); 2 Research Institute for Veterinary Science, College of Veterinary Medicine, Seoul National University, Seoul 88026, Korea; 3 Department of Microbiology and Immunology, Emory University School of Medicine, Atlanta, GA 30322, USA; Chung-Young.Lee@emory.edu; 4 KBNP, Inc., 235-9, Chusa-ro, Sinam-myeon, Yesan-gun 32417, Korea; nhlee21@kbnp.co.kr (N.-H.L.); yjjeong@kbnp.co.kr (Y.-J.J.); 5 Animal and Plant Quarantine Agency, Gimcheon-si 39960, Korea; happythomas@korea.kr (J.-G.C.); leeyj700@korea.kr (Y.-J.L.); greenkang@korea.kr (H.-M.K.); 6 Laboratory of Poultry Medicine, Department of Farm Animal Medicine, College of Veterinary Medicine and BK21 PLUS for Veterinary Science, Seoul National University, 1, Gwanak-ro, Seoul 88026, Korea; 7 Farm Animal Clinical Training and Research Center (FACTRC), GBST, Seoul National University, Seoul 88026, Korea

**Keywords:** avian influenza virus, H9N2, genetic evolution, mammalian non-pathogenicity, recombinant vaccine strain

## Abstract

For the development of an optimized Egyptian H9N2 vaccine candidate virus for poultry, various recombinant Egyptian H9N2 viruses generated by a PR8-based reverse genetics system were compared in terms of their productivity and biosafety since Egyptian H9N2 avian influenza viruses already possess mammalian pathogenicity-related mutations in the hemagglutinin (HA), neuraminidase (NA), and PB2 genes. The Egyptian HA and NA genes were more compatible with PR8 than with H9N2 AIV (01310) internal genes, and the 01310-derived recombinant H9N2 strains acquired the L226Q reverse mutation in HA after passages in eggs. Additionally, the introduction of a strong promoter at the 3′-ends of PB2 and PB1 genes induced an additional mutation of P221S. When recombinant Egyptian H9N2 viruses with intact or reverse mutated HA (L226Q and P221S) and NA (prototypic 2SBS) were compared, the virus with HA and NA mutations had high productivity in ECES but was lower in antigenicity when used as an inactivated vaccine due to its high binding affinity into non-specific inhibitors in eggs. Finally, we substituted the PB2 gene of PR8 with 01310 to remove the replication ability in mammalian hosts and successfully generated the best recombinant vaccine candidate in terms of immunogenicity, antigenicity, and biosafety.

## 1. Introduction

H9N2 avian influenza A viruses (AIVs) have been reported in human cases, and they act as internal gene donors for the generation of new highly pathogenic, reassortant H5N1 AIVs and low pathogenic H7N9 AIVs that cause poultry as well as human infections [1,2,3,4,5,6]. Since their first identification in chicken farms in South China in 1994, H9N2 viruses have spread to many countries in Asia and Africa and have differentiated into several (G1-like, Y280/G9-like, and Y439-like) lineages. The G1-like lineage further diverged into sublineages A, B, C, and D in Central Asia and the Middle East, and a unique reassortant of A and B sublineages became endemic in domestic poultry by 2010 in Egypt [7,8,9,10,11]. To date, four cases of human H9N2 infection and recent cases of human H5N1 infection have been reported in Egypt [12,13]. In addition, anti-H9N2 and anti-H5N1 antibodies were detected in domestic pigs in Egypt [14]. Therefore, the evolutionary steps and status of Egyptian H9N2 AIVs with cocirculation of H5N1 AIVs need to be investigated to further understand the potential risk of them becoming pandemic viruses.

G1-like H9N2 AIVs may have evolved in chickens, and the genetic constellation supporting efficient replication and transmission may have been selected. In the case of Y439-like H9N2 AIVs, passaging through embryonated chicken eggs (ECEs) resulted in the predominance of the NA stalk-deleted mutant strain and may reflect the importance of balancing hemagglutinin (HA) and neuraminidase (NA) activities. These phenomena have already been observed in natural H5Nx HPAIVs, and HA–NA balancing may be a key factor in viral fitness in chicken infections [15].

HA protein is a transmembrane glycoprotein exposed outside of virus particles, and the receptor binding site (RBS) in the globular head of HA binds to host cell receptors. Avian and human influenza A viruses (IAVs) have different preferences for sialic acids, which are α2,3-(α2,3SA) and α2,6-linked (α2,6SA) to galactose, and mutations in the RBS change receptor binding preferences and host specificity. The E190D and G225D mutations in the H1 subtype and the Q226L and G228S (H3 numbering) mutations in the H2 and H3 subtypes increase α2,6SA preference, and currently isolated H9N2 viruses already have Q226L mutations [16,17,18]. NA plays roles in virus release from infected cells and inhibits sialoproteins [19,20]. The NA tetramer has a cavity with catalytic activity in the globular head domain, and some NA subtypes (N1, N2, and N9) of AIVs have a second sialic acid binding site (2SBS) adjacent to the catalytic site [21]. The avian 2SBS (av2SBS) consists of three loops (370-loop, 400-loop, and 430-loop) and plays a role in the hemadsorption of NA, which is not conserved in human IAVs [22]. Interestingly, only the av2SBS of some H9N2 subtype AIVs has accumulated stepwise multiple mutations to decrease affinity to avian receptors, and it may increase viral fitness via HA–NA balancing with the corresponding HA [23].

The cooperation of surface HA and NA proteins with internal proteins may also be important for viral fitness, and the appearance and predominance of new reassortant AIVs in nature may reflect the importance of compatibility between surface and internal proteins as well as between internal proteins [24,25,26,27]. Although various cross-talks between viral proteins have been reported, viral fitness is directly and profoundly affected by polymerase activity [25,27]. PB2, PB1, and PA form a trimer and have coevolutionary relationships with each other. However, PB2 is considered to be crucial for host adaptation, and rank orders and accumulation patterns of mammalian pathogenicity-related mutations (MPMs) have been reported [28,29,30,31]. Early-acquired MPMs may be important to determine subsequent MPMs, and in contrast to D701N, the E627K mutation has successfully recruited multiple MPMs to become prevalent in human IAVs [31]. Egyptian H9N2 viruses have acquired somewhat different mutations, such as I292T, G590C, and E627V, from previous reports, and only E627V has been characterized to increase polymerase activity in mammalian cells [13,32,33]. Therefore, the effects of combined mutations on polymerase activity need to be addressed.

The commercial inactivated H9N2 AI vaccine for poultry has been extensively used in Egypt since 2012, but the dissimilarity of the vaccine strain with the field strain led to an antigenic drift in the antigenic site and escaping mutants [9,33,34,35]. There are inactivated vaccines using the field virus as it is or a recombinant virus containing only HA and NA genes of the wild-type virus. The preparation of inactivated field strain vaccines may be an easy choice, but handling live viruses infecting chickens and humans may be stressful in terms of biosafety and biosecurity. Conventional PR8-derived recombinant AI vaccine strains may be less pathogenic to chickens, but mammalian pathogenicity may be increased by the pathogenic factors in internal genes of PR8 [36,37]. Therefore, optimal methods to remove the mammalian pathogenicity of vaccine strains for biosafety while maintaining immunogenicity and antigenicity in poultry need to be selected.

In this study, we generated various recombinant Egyptian H9N2 viruses with different gene constellations to find the optimized recombinant vaccine strains using a 6 + 2 reverse genetics system. The compatibility of the HA and NA of Egyptian H9N2 AIVs with six internal genes of avian and human IAVs was tested, and the effects of mutations observed in the RBS of HA, 2SBS of NA, and PB2 of Egyptian H9N2 AIVs on viral fitness and their acquisition orders were investigated.

## 2. Materials and Methods

### 2.1. Viruses, Plasmids, Cells, and Eggs

Recombinant viruses were generated by a pHW2000 plasmid-based reverse genetics system provided by Dr. Robert Webster (St. Jude Children’s Research Hospital). Six internal genes (PB1, PB2, PA, NP, M, and NS) of A/Puerto Rico/8/1934 (H1N1) (PR8) and A/chicken/Korea/01310/2001 (H9N2) (E20, 01310) and the 3′ end promoter-mutated PB2 and PB1 genes of 01310 (01310-U4) were used in this study [38,39]. 293T, MDCK (Madin-Darby Canine Kidney), and A549 cells were purchased from Korean Collection for Type Cultures (KCTC) and maintained in DMEM supplemented with 10% fetal bovine serum (Life Technologies Co., Carlsbad, CA, USA) for transfection and the growth kinetics of recombinant viruses. Ten-day-old specific pathogen-free (SPF) embryonated chicken eggs (ECEs) (VALO BioMedia GmbH, Osterholz-Scharmbeck, Germany) were used for the propagation and titration of recombinant strains.

### 2.2. Sequence Analysis

The complete HA, NA, and PB2 genes of H9N2 AIVs were all collected from the GISAID Epiflu database, and the nucleotide sequences of HA and NA were aligned with the MEGA program. The nucleotide sequences were translated, and the amino acid mutations at specific residues were compared to calculate frequencies with the BioEdit program (v7.2.5).

### 2.3. Synthesis and Cloning of HA and NA Genes

HA and NA genes were synthesized according to the coding region of A/chicken/Egypt/ME543V/2016(H9N2) (accession no. MF434468.1, ME543), and the common 5′- and 3′-end noncoding region sequences of Egypt wild-type H9N2 isolates were determined from the GISAID database (Cosmo Genetech, Seoul, Korea). The prokaryotic promoters and ORFs in the H9 gene sequence were predicted with the Berkeley Drosophila Genome Project (BDGP) program. Site-directed mutagenesis was performed with a Muta-Direct™ site-directed mutagenesis kit (iNtRON) and primer sets according to the manufacturer’s instructions (Table S1). The HA and NA genes of ME543 were cloned into the pHW2000 plasmid as described previously [38].

### 2.4. Generation of Viruses by Reverse Genetics

293T cells were prepared in six-well plates one day in advance, and a mixture of each gene plasmid was transfected with Plus reagent and Lipofectamine 2000 (Life Technologies, Carlsbad, CA, USA) according to the supplier’s instructions. After one day of incubation at 37 °C in a CO_2_ incubator, 1 mL of Opti-MEM (Life Technologies) and 4 µg/well of L-1-tosylamido-2-phenylethyl chloromethyl ketone (TPCK)-treated trypsin (Sigma-Aldrich, St. Louis, MO, USA) were added and incubated for another 24 h. Two hundred microliters of cell supernatant was inoculated into 10 day old SPF ECEs (VALO biomedia GmbH) and incubated for 3 days. Virus passages through ECEs were repeated 3 or 4 times, and allantoic fluid was harvested and stored at −80 °C until use. The presence of virus was detected with a hemagglutination assay using 1% (*v*/*v*) chicken red blood cells (RBCs), and the full genome sequences were confirmed by RT-PCR and sequencing as previously described [40].

### 2.5. Determining Viral Infection Titer in ECEs

To measure the virus titer, each virus was serially diluted from 10^−1^ to 10^−9^ and inoculated into five 10 day old SPF ECEs for each dilution via the allantoic cavity route. After 3 days of incubation at 37 °C, the eggs were chilled at 4 °C overnight, and allantoic fluid was harvested. The presence of virus in each dilution was confirmed by a hemagglutination assay, and the EID_50_ of each virus was calculated by the Spearman–Karber method [41]. To compare replication efficiency in ECEs, 100 EID_50_/0.1 mL of each virus was inoculated into five 10 day old SPF ECEs, and virus titer was measured as above.

### 2.6. Receptor Binding Assays

To compare the receptor binding affinity of recombinant virus to α2,3-/α2,6-linked sialic acid, a solid-phase assay was used as previously described [42]. Briefly, 96-well enzyme-linked immunosorbent assay plates (SPL, Gyeonggi, Korea) were bound with 2^6^ HAU of recombinant viruses at 4 °C overnight. Virus-bound plates were washed with 0.1% PBST three times and blocked with PBS (Phosphate buffered saline) containing 1% desialylated BSA and 10 µM oseltamivir (Sigma-Aldrich) for 1 h at 4 °C. After plates were washed with PBST tgree times, serially three-fold diluted biotinylated sialylglycopolymers (Neu5Acα2-3Galb1-4GlcNAcb-PAA-biotin, 3′SLN-PAA, and Neu5Acα2-6GalNAca-PAA-biotin, 6′SLN-PAA) (Glycotech) were added and incubated for 1 h. The plates were washed again with PBST three times and reacted with horseradish peroxidase (HRP)-conjugated streptavidin (Thermo Fisher Scientific, Waltham, MA, USA) for 1 h. Finally, HRP was developed with 3,3′5,5′-tetramethylbenzidine (TMB) substrate (SurModics, Eden Prairie, MN, USA). Chromogenic reaction was stopped by adding 0.1 M sulfuric acid, and the absorbance at 450 nm was measured by a microplate reader (TECAN, Männedorf, Switzerland).

### 2.7. Neuraminidase Activity Test

The rEgH9N2(P), rEgH9N2(P)-av370L, and rEgH9N2(P)-av400L strains were diluted into 128 (2^7^) HAU at 4 °C to match the amount of virus in the absence of neuraminidase activity and used to compare the neuraminidase activities of each virus using the NA-Star™ Influenza Neuraminidase Inhibitor Resistance Detection Kit according to the manufacturer’s instructions (Thermo Fisher Scientific). Each virus was diluted five-fold with NA-Star™ assay buffer, and 50 μL of each dilution was transferred to a 96-well white plate in triplicate. The NA-Star™ substrate diluted 1:1000 with the assay buffer was added to every well and reacted at room temperature for 30 min. After the addition of 60 μL of the NA-Star™ accelerator and shaking for 2 s, luminescence was measured by Infinite 200 PRO (TECAN).

### 2.8. Heat and Low pH Stability Tests

Heat and low pH stability tests were conducted according to previously reported methods [36]. For the heat stability test, each virus was diluted into 32 (2^5^) HAU and aliquoted into seven 1.5 mL microcentrifuge tubes to incubate at 56 °C for 0, 0.5, 1, 1.5, 2, 3, and 4 h. After heat treatment, the HA titer of each aliquot was measured by a hemagglutination assay as described above. Low pH stability was tested in various pH buffer (5.0–5.6) conditions. Briefly, 10^7^ EID_50_ of each virus (50 μL) was mixed with 100 volumes of pH buffer (450 μL) at 37 °C for 1 h and neutralized by a 1/100 dilution with PBS (pH 7.4) before 10 day old ECE inoculation. After 3 days of incubation, the HA titer was measured.

### 2.9. Minigenome Assay

PB2 gene sequences of wild-type Egyptian H9N2 AIVs (*n* = 56) in the influenza database were collected and compared. Several mutation sites known or unknown as MPMs of PB2 were selected to confirm their effects on polymerase activities (Supplementary Table S2). Each mutation was introduced into the 01310 PB2 gene containing I66M, I109V, and I133V mutations (MVV) using a Muta-Direct™ site-directed mutagenesis kit and primer sets (Supplementary Table S1) [31]. Each PB2 gene plasmid (PR8 PB2, 01310 PB2, 01310 MVV-E627V, or 01310 MVV-all) was mixed with PR8 PB1, PR8 PA, PR8 NP, firefly luciferase, and pRL-TK plasmids. A mixture of plasmids was transfected into confluent 293T cells in a 24-well plate by Lipofectamine 3000 (Thermo Fisher Scientific) according to the manufacturer’s instructions. After 24 h of incubation at 37 °C in a CO_2_ incubator, the cells were washed once with PBS, and luciferase activity was measured using a Dual-Luciferase^®^ Reporter Assay System (Promega). Firefly luciferase activity was normalized to Renilla luciferase activity.

### 2.10. Virus Titration in Mammalian Cell Lines

The safety of recombinant Egypt H9N2 virus as an inactivated vaccine candidate was indirectly evaluated by infection in mammalian host-originated cell lines, Madin–Darby canine kidney (MDCK) cells, and A549 cells (KCTC). A total of 5 × 10^5^ EID_50_/0.5 mL of virus diluted in DMEM with 1 µg/mL TPCK-trypsin was inoculated into MDCK and A549 cells (5 × 10^5^ cells/mL) seeded in a 12-well plate for 1 h, and the inoculated virus was eliminated. Infected cells were washed twice with phosphate-buffered saline, and 1 mL of fresh medium was added for 3 days of incubation. Cell supernatant was obtained every 24 h (0, 24, 48, and 72 h), and the viral titer at each time point was measured at a 50% tissue culture infective dose (TCID_50_) through 10-fold diluent inoculation into MDCK cells prepared in 96-well plates and calculated with the Spearman–Karber method.

### 2.11. Preparation of Oil Emulsion Vaccines and Inoculation of Chickens

The inactivated recombinant Egyptian H9N2 vaccine efficacy test in chicken was approved and conducted in the facility at KBNP Inc. (IACUC no. KBNP 20-02). Inactivated oil emulsion vaccines were prepared with four recombinant Egypt H9N2 viruses: rEgH9N2(P) (K161ME), rEgH9N2(P)-310PB2 (K162ME), rEgH9N2(P)-P221S-L226Q-av2SBS (K163ME), and rEgH9N2(P)-P221S-L226Q-av2SBS-310PB2 (K164ME). Allantoic fluids containing each virus were inactivated by mixing with 0.2% formaldehyde (Sigma-Aldrich) and incubated at 37 °C for 24 h. The formaldehyde-treated virus was inoculated into 10 day old SPF ECEs to confirm virus inactivation, and confirmed viruses were mixed with ISA70 (SEPPIC) at a ratio of 3:7 to make an oil-emulsion vaccine. Ten one day old SPF chickens of each group were vaccinated with 0.2 mL of the oil-emulsion vaccine via the subcutaneous route. Serum from each group was collected at 0 and 3 weeks postvaccination (wpv) and used to measure serum antibody titers.

### 2.12. Hemagglutination Inhibition (HI) Test

Serum samples of each vaccinated group were treated at 56 °C for 30 min prior to serological testing to eliminate nonspecific hemagglutination inhibition reactions caused by proteins present in the serum, and the HI test was performed following the WHO Manual on Animal Influenza Diagnosis and Surveillance. Briefly, each serum sample was serially diluted two-fold with phosphate-buffered saline (PBS) and reacted with the same volume of 4 (2^2^) hemagglutination titer (HAT) of four vaccine strain antigens for 40 min. Then, 25 μL of 1% chicken RBCs was added, and serum antibody titer was recorded after 40 min of incubation.

### 2.13. Virus Purification, SDS-PAGE and Western Blotting

To compare the antigen quantity of vaccine candidate viruses in the same volume of allantoic fluid, the virus was purified using OptiPrep™ Density Gradient Medium (Sigma-Aldrich) and ultracentrifugation (Beckman Coulter, Brea, CA, USA). One hundred EID_50_/0.1 mL of each virus was inoculated into 10 day old SPF ECEs, and allantoic fluid was harvested after 72 h incubation at 37 °C. Nine milliliters of allantoic fluid was added to 7 mL of 10% OptiPrep medium in a thick-wall tube used for an SW 70 Ti rotor (Beckman Coulter) and centrifuged at 45,000 rpm (150,000× *g*) for 1 h. The sediment was eluted in 100 μL of sterilized water, and purified viral protein was deglycosylated with PNGase F (N-glycosidase F; New England Biolabs, Ipswich, MA, USA). Twenty microliters of total viral protein and degycosylated protein was treated with 4X Bolt™ LDS Sample Buffer (Thermo Fisher Scientific) for 5 min at 95 °C and separated with NuPAGE 4–12% Bis-Tris Protein Gels (Life Technologies Co.). The protein band was confirmed with Coomassie Brilliant Blue G-250 staining (Biosesang, Seongnam, Koera) for 1 h and destained with a solution consisting of 10% ethanol and 7.5% acetic acid. For IgY detection in purified virus, SDS-PAGE gels were transferred to nitrocellulose membranes. The transferred membrane was incubated with blocking buffer (5% skim milk) for 1 h at room temperature, and HRP-conjugated goat anti-chicken IgG (IgY) antibody (Thermo Fisher Scientific) (1:10,000 dilution) was added for 1 h after three washes with TBST. HRP was developed with BioFX TMB One Component HRP Membrane Substrate (SurModics).

### 2.14. Statistical Analyses

The significance of the results between experimental groups was evaluated by a one-way analysis of variance (95% confidence intervals, Bonferroni post-hoc test) (IBM SPSS statistics, Armonk, NY, USA), and *p* < 0.05 was defined as statistically significant.

## 3. Results

### 3.1. E. coli Toxic ORF Is Present in the In-Frame of the HA2 Coding Region of an Egyptian H9N2 Strain

**Figure 1:**
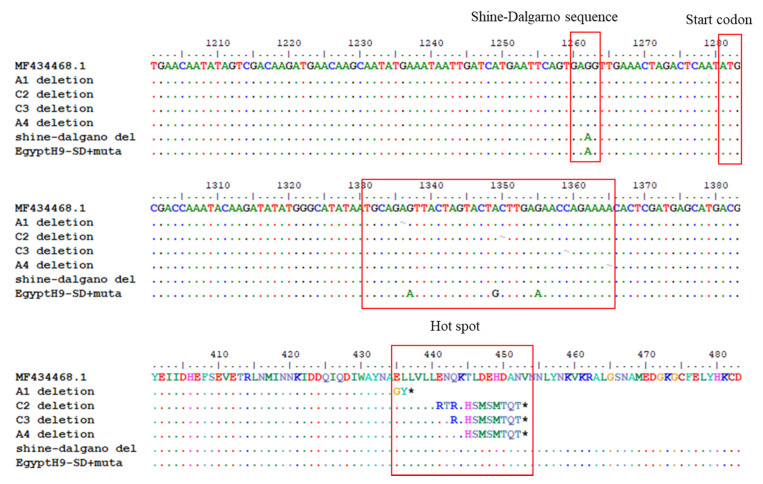
*E*. *coli* toxic ORF in the HA2 subunit gene and hotspots of deletion mutations. MF434468.1 was identical to the A/chicken/Egypt/ME543V/2016 (H9N2) (ME543), and synthetic H9 genes had a single nucleotide deletion (~) in four different positions of the hot spot (A at 1336, C at 1350, C at 1359 or A at 1365), forming a stop codon and terminating HA protein synthesis early. They encode the leucine-rich sequence (ELLVLL) and are mutated into the same nucleotide sequence of 01310 without amino acid change. Additionally, the Shine–Dalgarno sequence (GAGG) prior to the toxic ORF was eliminated by mutation into GAAG to reduce the expression. * means the stop codon and red box was site described with the text above it.

To make a recombinant H9N2 vaccine for the strain prevalent in Egypt, HA and NA genes coincident with the sequence of A/chicken/Egypt/ME543V/2016 (H9N2) (ME543) were used. In contrast to the NA gene sequence, the complete HA gene sequence could not be synthesized without a nucleotide (nt) deletion ranging from 1336 to 1365 (H9 numbering), which resulted in stop codons at amino acids 437 and 453 (Figure 1). Therefore, we predicted the *E. coli* promoter and open reading frames (ORFs) around the toxic regions and found the Shine–Dalgarno sequence (GAGG, 1260–1264) and the start codon (1281–1283). The GAGG sequence was mutated to GAAG, and transformed *E. coli* was incubated at low temperature (33 °C) to decrease the expression of the putative toxic gene. We successfully cloned the complete HA gene. Interestingly, the putative toxic ORF (144aa) had the same reading frame of HA2 of H9, but it had characteristics of outer-membrane proteins (OMPs) (Supplementary Table S4). The observed deletions caused the truncation not to form the second transmembrane region out of six predicted transmembrane regions.

### 3.2. Egyptian H9 and N2 Are Compatible with Mammalian (PR8) Internal Genes, but Incompatible Avian (01310) Internal Genes Induce the L226Q Reverse Mutation in HA

**Figure 2:**
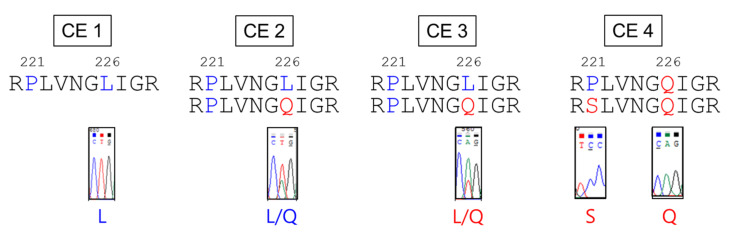
The six internal genes of 01310 induce reverse (L226Q) and additional (P221S) mutations in the HA of recombinant Egyptian H9N2 strains during ECEs passages. Recombinant Egypt H9N2 viruses with internal genes of 01310 were passaged to increase antigen yields in ECEs. All viruses showed increased replication efficiency in ECEs after the third passage by acquisition of the Q226L mutation in the HA protein, and rEgH9N2(310)-PB21U4 only had the additional P221S mutation in the HA protein after the fourth passage.

The A/Puerto Rico/8/1934 (PR8)-based Hoffmann’s bidirectional pHW2000 plasmid and reverse genetics systems were used for recombinant virus generation [38]. To increase the genetic homology of the full genomes with the wild-type H9N2 virus, six internal genes of H9N2 LPAIV A/chicken/Korea/01310/01 (H9N2) (01310) were also used. The PR8-derived recombinant Egypt H9N2 strain, rEgH9N2(P), grew well in 10 day old ECEs from the beginning, while rEgH9N2(310) with internal genes of 01310 showed low virus titers with a tendency to increase during passages (64 to 256 in HAU and 10^7.58^^±0.14^ to 10^8.53^^±0.70^ in EID_50_/mL, Table 1). Previously, we found that the mutation of the fourth nucleotide in the 3′-end of polymerase genes (PB2 and PB1) from cytosine (C4) to uracil (U4) increased the efficiency of recombinant virus rescue in a reverse genetics system [39]. To increase virus titers of 01310-derived recombinant strains in ECEs, we introduced a strong 3′-end promoter (U4) into the PB2 and/or PB1 genes of 01310 and generated rEgH9N2(310)-PB2U4, rEgH9N2(310)-PB1U4 and rEgH9N2(310)-PB21U4. As expected, the third (CE3) and fourth (CE4) passaged viruses with strong promoters showed higher virus titers than rEgH9N2(310) with weak promoters (C4). To shed light on the causes of increased virus titers of 01310-derived recombinant viruses, we determined the whole virus genome sequences to find a common L226Q mutation (H3 numbering) and a specific mutation (P221S) in only the HA gene of rEgH9N2(310)-PB21U4 (Table 1 and Figure 2). A quasispecies with the L226Q mutation began to appear from CE2 and became major (CE3) to predominant (CE4) during passages.

### 3.3. SBS Mutations in NA Appear Earlier than the L226Q Mutation in HA among H9N2 AIVs

**Figure 3:**
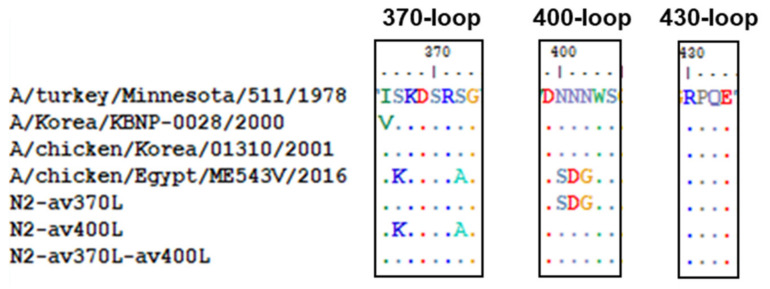
Comparison of the 2SBS amino acid sequences of H9N2 AIVs and mutated genes in this study. The 2SBS sequence of A/turkey/Minnesota/511/78(H9N2) was compared with others as the prototype. Three kinds of mutated NA genes were generated by replacing mutations of ME543V in the 370-loop (N2-av370L), the 400-loop (N2-av400L), or both (N2-av370L-av400L) with prototype sequences.

The frequencies of P221S and L226Q mutations in HA and 2SBS mutations in NA among H9N2 AIVs isolated during 1970–2020 are summarized in Table 2. All H9N2 AIVs (550 isolates) for which the HA and NA genes were available in the database were used for analysis. The Q226L mutation appeared first during 1991–2000 and became frequent at 86.5% during 2011–2020. However, none of the H9N2 AIVs acquired the P221S mutation. Since the single amino acid mutation in the 370-loop first appeared during 1960–1970, multiple mutations accumulated in the 370-loop (82.91%) and 400-loop (83.31%) during 2011–2020. Therefore, amino acid changes in 2SBS may have occurred earlier than the Q226L mutation and had a tendency to accumulate stepwise. Most Egyptian H9N2 AIVs already acquired the Q226L mutation and multiple amino acid changes in 2SBS (Supplementary Table S2). The 2SBS mutations of ME543V (EgN2) and mutant recombinant viruses generated in this study are compared in Figure 3.

### 3.4. Reverse Mutations in Only 2SBS of NA Decrease the Viral Fitness of Recombinant Strains

To investigate the effects of P221S, L226Q, av370L, and av400L mutations on virus replication in ECEs, PR8-derived recombinant Egyptian H9N2 strains were generated, as shown in Table 3. P221S and L226Q mutations did not cause differences in virus replication in ECEs, whereas av370L and av400L mutations slightly decreased virus titers. Interestingly, rEgH9N2(P)-av400L (CE3) with the av400L mutation acquired the L226Q mutation, and rEgH9N2(P)-av2SBS with the av370L and av400L mutations was not generated, suggesting that mutations in 2SBS critically affected virus survival. rEgH9N2(P)-P221S-av2SBS with P221S, av370L, and av400L mutations showed lower virus titers (10^8.07±0.06^ EID_50_/mL) than rEgH9N2(P) (10^9.42^^±0.38^ EID_50_/mL). However, rEgH9N2(P)-L226Q-av2SBS with L226Q, av370L, and av400L and rEgH9N2(P)-P221S-L226Q-av2SBS with L226Q, P221S, av370L, and av400L mutations showed higher (10^10.03 ± 0.06^ EID_50_/mL) and similar virus titers (10^9.37 ± 0.12^ EID_50_/mL), respectively (Table 1 and Table 3).

### 3.5. Mutations Reducing Avian Receptor Affinity Have No Effect on the Ratio of Avian to Mammalian Receptor Affinities

**Figure 4:**
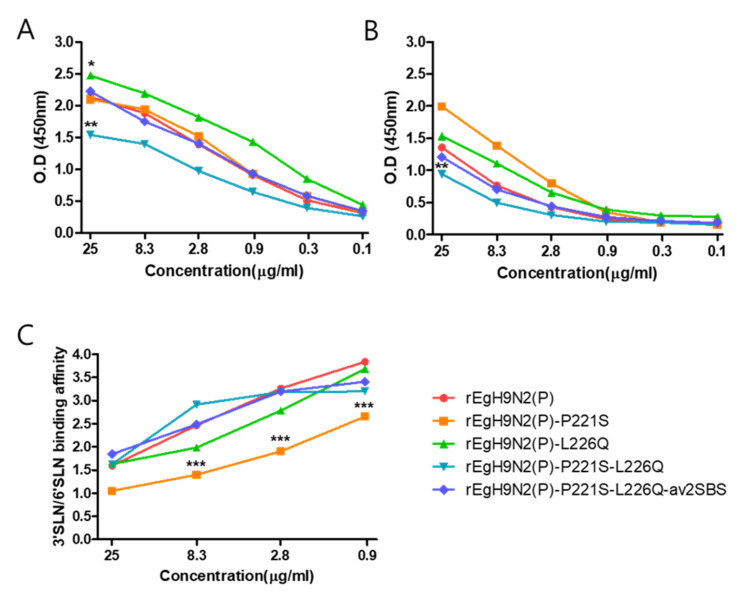
Most mutations decreasing avian receptor affinity have no effect on the ratio of avian to mammalian receptor affinities. Each virus was diluted to the same concentration (2^6^ HAU) with neuraminidase inhibitor, blocking buffer was added, and the virus was adsorbed on a 96-well immunoplate. (**A**) Avian receptor analog (3′SLN-PAA) and (**B**) human receptor analog (6′SLN-PAA) three-fold diluents were added and reacted with HRP-conjugated streptavidin after 1 h. HRP was developed by TMB, and the absorbance at 450 nm was measured after the addition of 0.1 M sulfuric acid. (**C**) The relative binding affinity was calculated by dividing the 3′SLN binding affinity by the 6′ SLN binding affinity, *: rEgH9N2(P)-L226Q had a significantly higher binding affinity to 3′SLN than other viruses, **: rEgH9N2(P)-P221S-L226Q showed significantly lower affinity to 3′SLN than other viruses and to 6′SLN than rEgH9N2(P)-P221S and rEgH9N2(P)-L226Q (*p* < 0.05), ***: rEgH9N2(P)-P221S was significantly different from other viruses (*p* < 0.05).

**Figure 5:**
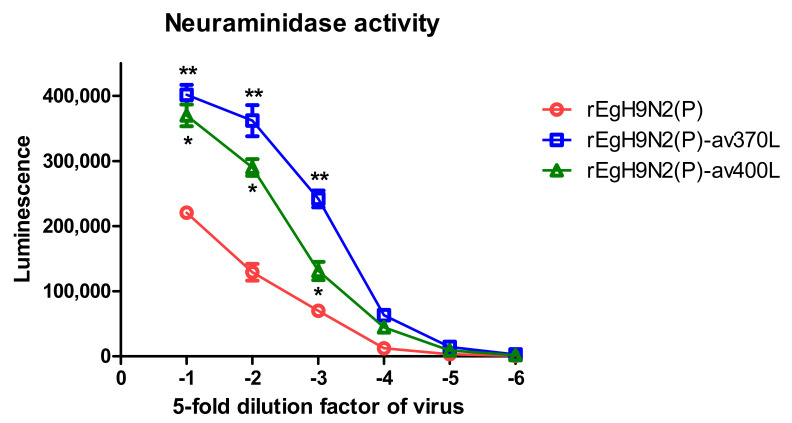
Reverse mutations in the 370-loop and 400-loop of NA increase neuraminidase activity via increased affinity to substrate. The 128(2^7^) HAU of each virus was diluted five-fold, and neuraminidase activity was measured in triplicate using the NA-star™ Influenza Neuraminidase Inhibitor Resistance Detection Kit. *, rEgH9N2(P)-av370L was significantly different from rEgH9N2(P); **, rEgH9N2(P)-av370L was significantly different from the others (*p* < 0.05).

rEgH9N2(P)-L226Q showed the highest affinity, but the additional P221S mutation (rEgH9N2(P)-P221S-L226Q) decreased the affinity to an avian receptor analog (3′SLN). However, the combination of all the mutations, L226Q, P221S, av370L, and av400L, (rEgH9N2(P)-P221S-L226Q-av2SBS) restored binding affinity (Figure 4A). In contrast to the others, rEgH9N2(P)-P221S showed relatively high affinity for the human receptor analog 6′SLN (Figure 4B). When comparing the avian to mammalian receptor affinity ratios (3′SLN/6′SLN), all tested recombinant strains showed higher affinity for 3′SLN than 6′SLN. Additionally, the ratios increased with decreasing concentrations of receptor analogs. Except for rEgH9N2(P)-P221S, the other recombinant strains did not show significant differences between their affinity ratios (Figure 4C). rEgH9N2(P)-av370L and rEgH9N2(P)-av400L showed significantly higher neuraminidase activities than rEgH9N2(P) and rEgH9N2(P) and rEgH9N2(P)-av370L, respectively (*p* < 0.05, Figure 5).

### 3.6. Egyptian H9 Is Relatively Heat-Stable and Has Evolved to Become Susceptible to Low pH

**Figure 6:**
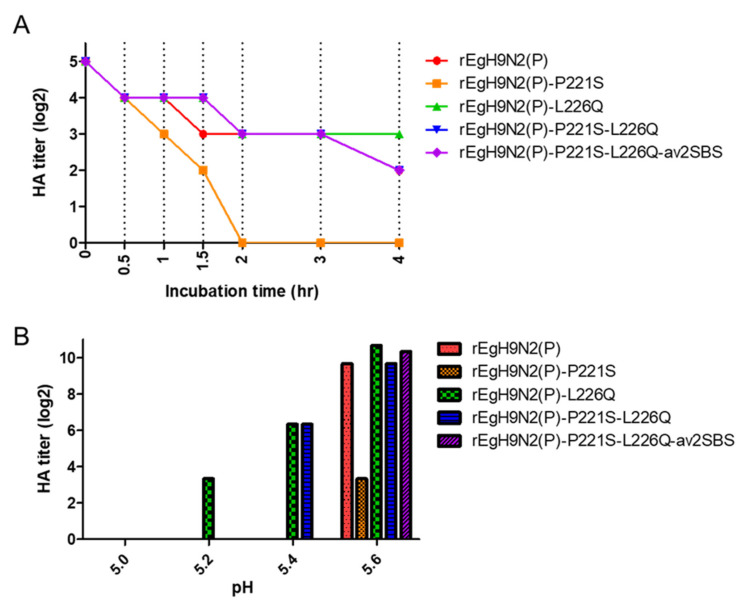
Relatively high heat stability and low pH susceptibility of recombinant Egyptian H9N2 strains. (**A**) Each recombinant virus was diluted to 32 (2^5^) HAU and incubated at 56 °C for 0.5, 1, 1.5, 2, 3, and 4 h. After heat treatment, the HA titer of each aliquot was measured and recorded. (**B**) A total of 10^7^ EID_50_ of viruses was mixed with pH buffer (5.0–5.6) at a 1:100 ratio and incubated at 37 °C for 1 h. Mixtures were diluted with PBS (pH 7.4) at a 1:100 ratio and inoculated into three 10 day old ECEs. The HA titers of viral replicates in harvested allantoic fluid after 72 h were compared.

In contrast to rEgH9N2(P)-P221S, the HA titers of the remaining strains gradually decreased to maintain 2^2−3^ HAU after heat treatment at 56 °C for 4 h (Figure 6A). The L226Q mutation tended to increase heat stability, while P221S tended to reduce the heat stability of the HA protein singly and in combination with other mutations. In the same context, the L226Q mutation tended to increase acid stability to maintain HA activity even at pH 5.2 [rEgH9N2(p)-L226Q], while P221S tended to reduce acid stability even at pH 5.6 [rEgH9N2(P)-P221S]. Additionally, av370L and av400L mutations tended to decrease acid stability at pH 5.4 compared with rEgH9N2(P)-P221S-L226Q and rEgH9N2(P)-P221S-L226Q-av2SBS (Figure 6B).

### 3.7. The E627V Mutation Is a Key MPM including the I292T, K526R, G590C, and S714G Mutations

**Figure 7:**
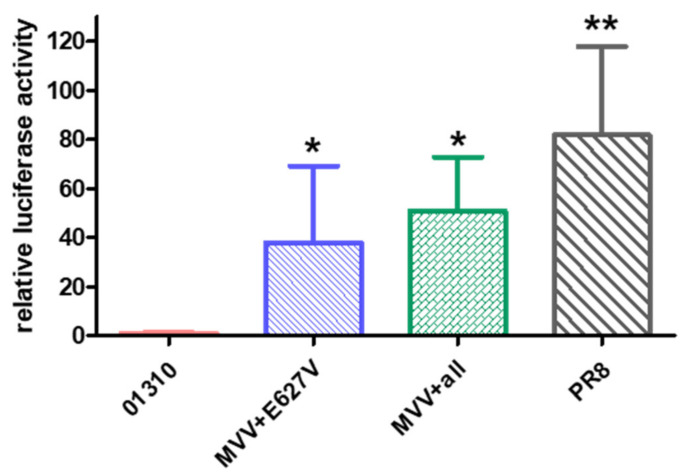
The E627V mutation of PB2 is a key MPM of Egyptian H9N2 AIVs. Based on the plasmid encoding PB2 of 01310 with I66M, I109V, and I133V mutations (MVV), single (E627V) and multiple (I292T, K526R, G590C, E627V, and S714G) mutations acquired by Egypt H9N2 viruses were introduced. Each PB2 plasmid was cotransfected with plasmids for PB1, PA, NP of PR8, firefly luciferase, and pRL-TK plasmid into 293T cells. Firefly luciferase activity was normalized to Renilla luciferase activity and compared with the PB2 of PR8. The average and standard deviation of three independent replicates are shown. * Significantly different from the PB2 gene of 01310, ** Significantly different with others (*p* < 0.05).

Egyptian H9N2 AIVs already had I66M, I109V, and I133V mutations (MVVs) in their PB2 genes, which may be a prerequisite for additional MPM acquisition, and they cumulatively acquired additional mutations (I292T, K526R, G590C, E627V, and S714G) (Supplementary Table S3) [31,32]. We compared the effects of the key mutation, E627V, and all the combined mutations on polymerase activity (Figure 7). In combination with MVV, the E627V mutation significantly increased polymerase activity compared with 01310 and was comparable with all combined mutations with additional mutations (I292T, K526R, G590C, and S714G). However, the polymerase activity of the rPR8 polymerase trimer was significantly higher than that of the other polymerases.

### 3.8. The 01310 PB2 Gene Is Compatible with Egyptian H9 and N2 Genes

To date, the 01310 PB2 gene has been successfully used for the development of AI vaccine strains in terms of high virus titers, strong immunogenicity, and mammalian nonpathogenicity [36,37,40]. Therefore, we generated recombinant strains with various combinations of mutations (Table 4). rEgH9N2(P)-310PB2-av2SBS and rEgH9N2(P)-310PB2-P221S-av2SBS were not rescued, and the results were reminiscent of the failure of rEgH9N2(P)-av2SBS rescue and relatively low virus titer of rEgH9N2(P)-P221S-av2SBS. rEgH9N2(P)-310PB2 and rEgH9N2(P)-310PB2-P221S-L226Q-av2SBS showed higher virus titers than the corresponding rEgH9N2(P) and rEgH9N2(P)-P221S-L226Q-av2SBS strains, respectively, in ECEs without statistical significance (*p* > 0.05, Table 4). With the addition of the L226Q mutation, rEgH9N2(P)-310PB2-L226Q-av2SBS was rescued but showed lower virus titers than rEgH9N2(P)-310PB2 and rEgH9N2(P)-310PB2-P221S-L226Q-av2SBS.

### 3.9. The Six Internal Genes of PR8 Facilitate While the 01310 PB2 Gene Completely Attenuates Replications of Recombinant Egyptian H9N2 Strains in Mammalian Cells

**Figure 8:**
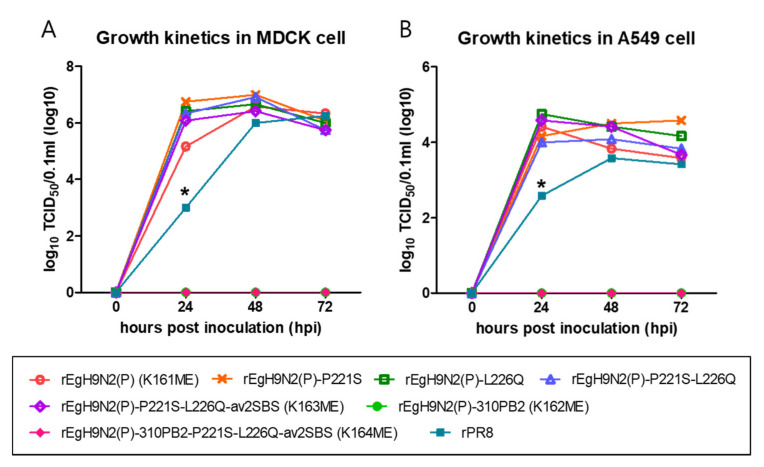
More efficient replication of PR8-derived recombinant Egyptian H9N2 strains than rPR8 and their attenuation by 01310 PB2 in mammalian cells. A total of 5 × 10^5^ EID_50_ (1 MOI) of each virus was inoculated into (**A**) MDCK and (**B**) A549 cells prepared in 12-well plates. After 1 h of incubation at 37 °C, the cells were washed with PBS, and 1 mL of fresh medium was added to each well. During 72 h of incubation, supernatants were obtained at 24, 48, and 72 h, and viral titers in supernatants were measured in TCID_50_. * The amount of rPR8 was significantly lower than that of other recombinant H9N2 viruses (*p* < 0.05).

The potential mammalian pathogenicity of AIVs can be predicted by replication efficiency in MDCK and A549 cell lines. In particular, A549 is an in vitro model that is in good correlation with the BALB/c mouse model [43]. The growth kinetics of recombinant Egyptian H9N2 strains that showed high virus titers in ECEs were investigated in MDCK and A549 cells (Figure 8). rEgH9N2(P)-310PB2 and rEgH9N2(P)-310PB2-P221S-L226Q-av2SBS possessing 01310 PB2 did not replicate in MDCK and A549 cells at any time point, as expected. However, all Egyptian H9N2 strains with the PR8 PB2 gene replicated efficiently enough to reach significantly higher titers than rPR8 at 24 h post inoculation (hpi). Therefore, reverse mutations to increase avian receptor affinities of HA and NA did not reduce viral replication efficiency in MDCK and A549 cells.

### 3.10. The Recombinant Egyptian H9N2 Strain Possessing Parent HA and NA and 01310 PB2 Genes Is Best in Immunogenicity and Antigenicity

Four Egyptian H9N2 strains, rEgH9N2(P) (briefly K161ME), rEgH9N2(P)-310PB2 (K162ME), rEgH9N2(P)-P221S-L226Q-av2SBS (K163ME), and rEgH9N2(P)-P221-L226Q-av2SBS-310PB2 (K164ME), were selected to prepare formalin-inactivated oil emulsion vaccines and compare their immunogenicities and antigenicities in chickens (Table 5). All of them showed sufficiently high immunogenicity to induce high HI titers above 512 (2^9^) at 3 weeks postvaccination (wpv), but K16E had the highest HI titers to homologous and heterologous strains. Interestingly, K163ME and K164ME with HA and NA mutations showed relatively low HI titers to homologous and heterologous strains.

### 3.11. Recombinant Egyptian H9N2 Strains Possessing Mutations Increasing Avian Receptor Affinity Bind More Sialoproteins in the Allantoic Fluid

**Figure 9:**
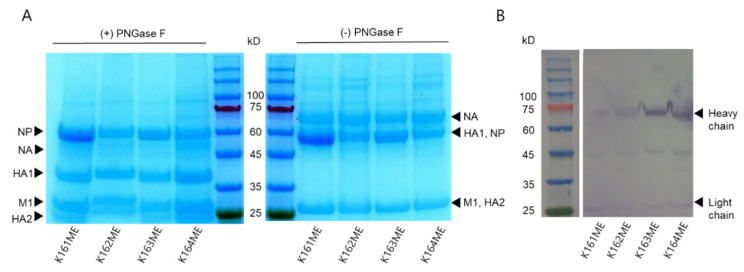
Different amounts of viral proteins and interactions with sialoproteins in allantoic fluids. The harvested allantoic fluid of SPF ECEs infected with vaccine candidate viruses was mixed with OptiPrep density gradient medium and ultracentrifuged at 45,000 rpm for 1 h. (**A**) Total viral protein purified and deglycosylated by PNGase F of vaccine candidate viruses was separated by SDS-PAGE and stained with Coomassie blue. (**B**) Separated protein-transferred membranes were blocked with 5% skim milk and treated with HRP-conjugated goat anti-chicken IgG (IgY) antibody for 1 h. HRP was developed with TMB solution, and chicken IgY was confirmed.

To understand why the four inactivated vaccines showed different immunogenicities and antigenicities, purified viral proteins were compared by SDS-PAGE, and the quantity of separated protein was measured using ImageJ 1.53a. Interestingly, the amounts of NP (PNGase F-treated) and NP + HA1 (PNGase-untreated) were higher in K161ME and K163ME than in K162ME and K164ME (Figure 9A). K164ME showed slightly higher amounts of HA1 than the others, but K161ME, K162ME, and K163ME showed similar amounts. During the purification of AIVs in allantoic fluid, nonspecific IgY coprecipitates with virus particles. The presence of coprecipitated IgY was determined by Western blotting after SDS-PAGE, and interestingly, more IgY was detected in K163ME and K164ME than in K161ME and K162ME (Figure 9B). This means that the higher affinity of mutated HA and NA to avian receptors increased the coprecipitation of IgY bearing four N-linked glycans.

## 4. Discussion

To date, various HA genes of AIVs have been cloned into *E.* *coli* plasmids, and our cloning failure was unexpected. HA gene cloning using *E. coli* after gene synthesis or amplification is the starting point of reverse genetics, and the problem needs to be solved. To our knowledge, the identification of toxic fragments in HA2 to *E. coli* has rarely been reported. We analyzed various H9N2 HA genes recently deposited in the database, but we could not find similar putative *E. coli* ORFs (data not shown). Although this may be an uncommon case, we can solve similar problems when we confront them in the future.

The low compatibility of Egyptian HA and NA genes with six 01310 internal genes, which supported the high growth of 01310E20, resulted in decreased replication efficiency but might have been a good condition for the outgrowth of quasispecies that acquired mutations, improving replication efficiency. The commonly shared reverse mutation L226Q may be the best way to recover the replication efficiency of recombinant strains with incompatible internal genes and the NA gene encoding low affinity to avian receptors [22,23,44,45]. Considering the earlier appearance of mutations in 2SBS of NA than in Q226L and the acquisition of the reverse L226Q mutation without a change in 2SBS, H9N2 AIVs may have evolved to acquire traits, first reducing the avian receptor affinity of NA, resulting in reduced enzyme activity compared with that of HA. This notion was also supported by the lack of virus rescues [rEgH9N2(P)-av2SBS and rEgH9N2(P)-310PB2-av2SBS] with wild-type HA and reversely mutated NA (av2SBS) (Table 3 and Table 4). Stepwise mutation acquisition in NA and HA reflects the balancing of their activities for viral fitness. However, why Egyptian H9N2 AIVs did not reduce NA activity through the large deletion of the NA stalk, which is frequently observed in other AIVs, is unknown [46,47,48].

The strong promoters in PB2 and PB1 genes induced an additional HA mutation, P221S, and it may contribute to the outgrowth of the quasispecies among only the L226Q-bearing viral population in ECEs. The P221S mutation has been reported in H5N1 AIVs to reduce HA trimer integrity to cause low heat and acid stabilities [49,50]. The P221S mutation among H9N2 AIVs is seemingly not natural; it may induce early viral envelope and endosome fusion due to low pH stability and speed up virus replication cycles [40,51]. Interestingly, the lower heat and acid stabilities and avian-to-mammalian-receptor-affinity ratio did not hinder virus replication in MDCK and A549 cells. The relatively high heat stability of wild-type Egyptian HA may help with virus survival in the environment, but low pH stability, representing a high activation pH, may not be favorable for human-to-human transmission [52,53]. Therefore, this may be one of the reasons for the lack of reports on human-to-human transmission in Egypt.

In this study, the higher compatibility of six PR8 internal genes with Egyptian than its own HA and NA genes was clearly demonstrated by the growth kinetics study in mammalian cells (Figure 8). Although we did not use the whole PB2 gene of Egyptian H9N2 virus for the polymerase assay, the combined mutations of Egyptian PB2 may not confer higher polymerase activity than rPR8 PB2. The E627K mutation is the most potent mutation for increasing polymerase activity in mammalian cells, and it is the most favorable early mutation for recruiting almost all MPMs [43]. The effect of the E627V mutation in Egyptian H9N2 virus on polymerase activity has been reported, but it may be less than that of the E627K mutation [32]. Although we did not directly compare the effects of Egyptian and PR8 genes, our result demonstrating that Egyptian PB2 is less pathogenic to mammals than PR8 PB2 genes may be a reasonable speculation (Figure 8). Therefore, the generation of a PR8-derived Egyptian H9N2 vaccine strain, if possible, needs to be avoided with respect to the biosafety of vaccine production procedures.

Egyptian H9N2 AIVs have evolved for more than 10 years in chickens, with some occasional infections in pigs and humans, and it is unclear in which host animals they acquire MPMs, especially the E627V mutation [12,54]. For the E627V mutation, transversion is required, and it might occur more rarely than transition for the E627K mutation [32]. The E627K mutation was not favorable for replication in avian hosts, and the prevalence of Egyptian H9N2 AIVs with E627V among chickens may reflect its neutral or nonnegative trait for replication in both hosts (Supplementary Table S3). However, the culling of pigs kept in open yards and fed on refuse in 2009, the recovery of the pig population in Egypt since 2011, and the prevalence of anti-H9 antibody in pigs should be noted to understand the appearance of the E627V mutation since 2011 in Egypt [14]. In addition, the Q591K mutation, which was acquired during mammalian infection, may reflect various ways of ongoing mammalian adaptation (Supplementary Table S3) [55].

To date, the gain-of-function of viruses has been a concern for a long time due to the potential risks of laboratory and manufacturing leaks of viruses, but why the generation of PR8-derived vaccine strains against mammalian pathogenic AIVs has not been discussed in biosafety and biosecurity aspects in depth is strange [56]. In our previous studies, the simple exchange of PR8 PB2 with 01310 PB2 genes attenuated the mammalian pathogenicity of AIVs with maintenance of virus replication efficiency in ECEs and immunogenicity [36,40]. In this study, we verified the usefulness of 01310 PB2 again, but our additional intention to reduce mammalian pathogenicity and increase virus replication efficiency in ECEs by reversing mutations in HA and NA was proven to be ineffective.

The immunogenicity and antigenicity of the whole virus vaccine depend on the antigen amount and epitope similarity, respectively. The highest HI antibody titers of rEgH9N2(P)-310PB2 against homologous and heterologous antigens need to be addressed (Table 5). Although the virus titers of vaccine candidate strains were similar, the protein contents were different. The lower and similar amounts of NP and HA1 of rEgH9N2(P)-310PB2 in comparison with rEgH9N2(P), respectively, and the higher amounts of coprecipitated IgY with recombinant strains with higher HA and NA affinity to avian receptors may be interesting observations (Figure 9). To date, several proteins affecting protein contents in virions have been reported, but the role of PB2 has rarely been studied [24,25]. During formalin inactivation, coprecipitated IgY may be cross-linked to HA and NA proteins via covalent bonds. Usually, immune complexes of antigens and antibodies are known to potentiate early humoral immunity [57]. However, the binding of IgY and other sialoproteins in allantoic fluid to the RBS of HA and 2SBS of NA might not increase the immunogenicity and antigenicity of K163ME and K164ME. Thus, the mutations related with host adaptation should be mutated with deliberation when producing avian influenza vaccine strains.

## 5. Conclusions

In this study, recombinant Egyptian H9N2 vaccine candidate viruses with different genetic characteristics were generated using a plasmid-based reverse genetics system, and we compared the productivity in ECEs, the mammalian pathogenic risk, and the antigenicity as a poultry vaccine. A PR8-derived recombinant vaccine strain possessing parent HA and NA genes and the 01310 PB2 gene was successfully selected as the best vaccine candidate against Egyptian H9N2 AIVs undergoing mammalian adaptation in terms of immunogenicity, antigenicity, and mammalian nonpathogenicity.

## Figures and Tables

**Table 1:** Genomic compositions and virus titers of recombinant Egyptian H9N2 strains.

Recombinant Virus	PB2	PB1	PA/NP/ M/NS	HA Titer			EID_50_/mL		Acquired HA Mutation
				CE2	CE3	CE4	CE3	CE4 ^a^	
rEgH9N2(P) ^b^	PR8	PR8	PR8	512	512	512	9.42 ± 0.38	9.42 ± 0.29	None
rEgH9N2(310)	01310	01310	01310	64	64 *	256 *	7.58 ± 0.14	8.53 ± 0.40	L226Q
rEgH9N2(310)-PB2U4	01310-U4 ^c^	01310	01310	64	64 *	128 *	8.55 ± 0.33	9.63 ± 0.12	L226Q
rEgH9N2(310)-PB1U4	01310	01310-U4 ^c^	01310	64	128 *	512 *	8.90 ± 0.17	8.90 ± 0.20	L226Q
rEgH9N2(310)-PB21U4	01310-U4	01310-U4	01310	64	256 *	512 *^,†^	8.60 ± 0.26	9.03 ± 0.31	P221S L226Q

^a^ EID_50_/mL of harvested virus (CE4) after 100 EID_50_ of third passage virus (CE3 virus) inoculation into 10 day old SPF ECEs. Average ± standard deviation of three independent replicates. ^b^ H9 and N2 gene sequences had identical coding regions as A/chicken/Egypt/ME543V/2016 (H9N2). ^c^ The fourth nucleotide in the 3′-end noncoding region was mutated from C (C4) to U(U4). * The major amino acid at position 226 (H3 numbering) was mutated from leucine to glutamine (L226Q). ^†^ The major amino acid at position 221 (H3 numbering) was mutated from proline to serine (P221S).

**Table 2:** Frequency of identified amino acid changes in HA and NA of H9N2 AIVs in each time period.

Isolation Period	Number of Sequences	Frequency of HA Mutation (%)		Frequency of NA Mutation ^b^ (%)		
		P221S ^a^	Q226L	370-Loop	400-Loop	430-Loop
1960–1970	2	0.0	0.0	50.0	50.0	0.0
1971–1980	10	0.0	0.0	10.0	20.0	0.0
1981–1990	13	0.0	0.0	23.1	0.0	0.0
1991–2000	57	0.0	21.1	82.8	77.6	8.6
2001–2010	194	0.0	49.9	80.4	81.4	20.6
2011–2020	274	0.0	86.5	82.9	83.3	14.5
total	550	0.0	62.9	79.2	78.8	15.4

^a^ H3 numbering. ^b^ Any amino acid changes in the prototypic amino acid sequences of 370-loop (ISKGSRSG), 400-loop (DNNNWS), and 430-loop (RPQE) were counted as mutations.

**Table 3:** Genomic compositions and virus titers of PR8-derived recombinant Egyptian H9N2 strains.

Recombinant Virus	HA	NA	Internal Genes	EID_50_/mL	
				CE2	CE3 ^a^
rEgH9N2(P)-P221S	H9-P221S	N2	PR8	8.67 ± 0.00	9.25 ± 0.43
rEgH9N2(P)-L226Q	H9-L226Q	N2	PR8	8.67 ± 0.00	9.25 ± 0.25
rEgH9N2(P)-P221S-L226Q	H9-P221S-L226Q	N2	PR8	8.92 ± 0.35	9.17 ± 0.58
rEgH9N2(P)-av370L	H9	N2-av370L	PR8	8.42 ± 0.29	9.00 ± 0.00
rEgH9N2(P)- av400L	H9	N2-av400L	PR8	8.25 ± 0.25	8.82 ± 0.39 *
rEgH9N2(P)-av2SBS	H9	N2-av370L-av400L	PR8	nd ^b^	nd
rEgH9N2(P)-P221S-av2SBS	H9-P221S	N2-av370L-av400L	PR8	8.25 ± 0.25	8.07 ± 0.06 ^†^
rEgH9N2(P)-L226Q-av2SBS	H9-L226Q	N2-av370L-av400L	PR8	8.83 ± 0.14	10.03 ± 0.06
rEgH9N2(P)-P221S-L226Q-av2SBS	H9-P221S-L226Q	N2-av370L-av400L	PR8	8.92 ± 0.14	9.37 ± 0.12

^a^ EID_50_/mL of harvested virus (CE3) after 100 EID_50_ of second passage virus (CE2 virus) inoculation into 10 day old SPF ECEs. Average ± standard deviation of three independent replicates. ^b^ not detected. * The major amino acid at position 226 (H3 numbering) was mutated from lysine to glutamine. ^†^ Significantly different from other viruses except for rEgH9N2(P)-370L and 400L (*p* < 0.05).

**Table 4:** PR8-derived recombinant Egyptian H9N2 strains with the 01310 PB2 gene and viral titers in ECEs.

Recombinant Virus	HA	NA	PB2	Internal Genes	EID_50_/mL	
					CE2	CE3 ^a^
rEgH9N2(P)-310PB2	H9	N2	01310	PR8	9.00 ± 0.25	10.08 ± 0.14
rEgH9N2(P)-310PB2- av2SBS	H9	N2-av370L-av400L	01310	PR8	nd ^b^	nd
rEgH9N2(P)-310PB2- P221S-av2SBS	H9-P221S	N2-av370L-av400L	01310	PR8	nd	Np ^c^
rEgH9N2(P)-310PB2- L226Q-av2SBS	H9-L226Q	N2-av370L-av400L	01310	PR8	8.67 ± 0.14	9.30 ± 0.35
rEgH9N2(P)-310PB2- P221S-L226Q-av2SBS	H9-P221S-L226Q	N2-av370L-av400L	01310	PR8	8.50 ± 0.25	9.92 ± 0.38

^a^ EID_50_/mL of harvested virus (CE3) after 100 EID_50_ of second-passage virus (CE2 virus) inoculation into 10 day old SPF ECEs. Average ± standard deviation of three independent replicates. ^b^ Not detected. ^c^ Not passaged.

**Table 5:** Serum antibody titers of chickens vaccinated with inactivated oil emulsion of recombinant Egypt H9N2 strains.

Recombinant Virus	Inactivated Vaccine Name	GMT of HI Titer ^a^			
		Antigen			
		K161ME	K162ME	K163ME	K164ME
rEgH9N2(P)	K161ME	1522 (1080–2144)	1131 (887.3–1441)	861.1 (571.6–1297)	1579 (1170–2132)
rEgH9N2(P)-310PB2	K162ME	2756 * (1957–3883)	2233 * (1258–3967)	939.0 (578.9–1523)	2233 * (1377–3623)
rEgH9N2(P)-P221S, L226Q/av2SBS	K163ME	624.1 (384.4–1013)	608.9 (364.3–1018)	558.3 (344.2–905.6)	789.6 (585.0–1066)
rEgH9N2(P)-P221S, L226Q/av2SBS-310PB2	K164ME	760.8 (459.4–1260)	724.1 (531.2–987.0)	558.3 (344.2–905.6)	939.0 (765.0–1153)

^a^ Geometric mean HI titer with 95% confident interval. * Significantly different from other vaccinated groups (*p* < 0.05).

## Supplementary Materials

The following supporting information can be downloaded at https://www.mdpi.com/article/10.3390/vaccines10020162/s1: Table S1: Primers used in the cloning and mutagenesis of HA and NA genes; Table S2: Mammalian pathogenic mutation sites in PB2 of Egypt H9N2 viruses; Table S3: Step-wise accumulation of mutations in 2SBS of N2; Table S4: Predicted prokaryote promoter in H9 sequence of H9N2 vaccine strain using Berkeley Drosophila Genome Project (BDGP) program; Figure S1: Original unedited Figure 9A,B. Intensity of each bands were measured using ImageJ (https://imagej.nih.gov/ij/download.html) program and the results were indicated near each bands.
