# Supplementary material for: Selection of an Optimal Recombinant Egyptian H9N2 Avian Influenza Vaccine Strain for Poultry with High Antigenicity and Safety
Source: Vaccines (Basel). 2022 Jan 21;10(2):162. doi: 10.3390/vaccines10020162 (PMC8876024; doi:10.3390/vaccines10020162)
Supplement: Supplementary file 1 [file vaccines-10-00162-s001.zip › vaccines-1548796-supplementary.pdf]

Table S1. Primers used in the cloning and mutagenesis of HA and NA genes

Primer name	Sequences (5'- 3')
EgH9-SD-F	CATGAATTCAGTGAAGTTCAAACTAGACTC
EgH9-SD-R	GAGTCTAGTTTGAACTTCACTGAATTCATG
EgH9-P221S-F	TTGATAGGGCCAAGGTCCCTTGTCAATGGTCT
EgH9-P221S-R	AGACCATTGACAAGGGACCTTGGCCCTATCAA
EgH9-L226Q-F	CCTTGTCAATGGTCAGATTGGGAGAATTAA
EgH9-L226Q-R	TTAATTCTCCCAATCTGACCATTGACAAGG
EgN2-370L-F	TTGGATGGGAAGAACAATCAGCAAGGATTCACGCTCAGGT
EgN2-370L-R	CAATGACCTTGAAAGTCTCATAACCTGAGCGTGAATCCTTGCTGAT
EgN2-400L-F	GGCAAGTCATAGTTGACAATAACAACTGGTCT
EgN2-400L-R	GAGAATATACCAGAATACCCAGACCAGTTGTTATTGTC
PB2-I292T-F	CACAAATAGGCGGAACAAGAATGGTAGACA
PB2-I292T-R	TGTCTACCATTCTTGTTCCGCCTATTTGTG
PB2-K526R-F	CACAAGGGACGGAACGGTTAACAATAACAT
PB2-K526R-R	ATGTTATTGTTAACCGTTCCGTCCCTTGTG
PB2-G590C-F	TAAAGCTGCCAGATGCCAATATAGTGGATT
PB2-G590C-R	AATCCACTATATTGGCATCTGGCAGCTTTA
PB2-E627V-F	ATTTGCCGCAGCCCCACCAGTGCAGAGTAGGATGCAGTTTT
PB2-E627V-F	AAAACTGCATCCTACTCTGCACTGGTGGGGCTGCGGCAAAT
PB2-S714G-F	AGCATCAACGAATTGGGCAATCTTGCGAAA
PB2-S714G-R	TTTCGCAAGATTGCCCAATTCGTTGATGCT

Table S2. Mammalian pathogenic mutation sites in PB2 of Egypt H9N2 viruses

Genotype	66	109	133	292	526	590	591	627	714	n.	Strain	Accession number
PB2-P	I	I	I	I	K	G	Q	E	S	0	A/chicken/Korea/01310/01	JX094853
PB2-M-0	M	V	V	I	K	G	Q	E	S	3	A/avian/Egypt/920431/2006	GU050309
PB2-M-1	M	V	V	I	K	G	K	E	S	2	A/pigeon/Egypt/S10409A/2014	KX000764
PB2-M-2	M	V	V	V	K	G	Q	E	S	9	A/chicken/Egypt/ABD1/2015	MG706981
PB2-M-3-1	M	V	V	T	R	G	Q	V	G	22	A/chicken/Egypt/S4456B/2011	JX273136
PB2-M-3-2	M	V	V	T	R	C	Q	V	G	18	A/chicken/Egypt/D7108E/2013	KF881331

Table S3. Step-wise accumulation of mutations in 2SBS of N2

Genotype	370L	400L	Strain	Accession no
P	ISKDSRSG	DNNNWS		
M-0-1	--------	--D---	A/chicken/Hidalgo/1433-6/2005(H5N2)	AIR94075
M-1-2	-K------	-SD---	A/sparrow/Guangxi/121/2007(H9N2)	ADC97094
M-1-3	-K------	-SDS--	A/quail/Egypt/14864V/2014(H9N2)	APF29585
M-2-1	-K----A-	--D---	A/chicken/Israel/1033/2007(H9N2)	ACJ68767
M-2-2	-K----A-	-SD---	A/chicken/Egypt/Q18036B/2019(H9N2)	QOM99314
M-2-3	-K----A-	-SDS--	A/quail/Egypt/113413v/2011(H9N2)	AER34920
M-3-1	-K--L-A-	--D---	A/Layers/Senegal/17VIR4455-2/2017(H9N2)	QJX58839
M-4-1	-KR-L-A-	--D---	A/chicken/Egypt/141794V/2014(H9N2)	APF29586

Promoter predictions for seq0 :			
**Start**	**End**	**Score**	**Promoter Sequence**
27	72	0.96	AGCAAAATGGAAATAATACCACTGATGACTATGCTGTTACTAGTGACAAC
291	336	1	ATGTTGTTGGGGGGAAGGGAATGGTCCTATATCGTTGAAAGACCATCAGC
320	365	0.86	TATCGTTGAAAGACCATCAGCAGTGAATGGAACATGTTACCCTGGGAATG
365	410	0.99	GAATGTGGAAAACTTAGAGGAACTCAGAATACTTTTTAGTTCCTCTAGTT
393	438	0.84	ATACTTTTTAGTTCCTCTAGTTCATATCAAAGAATTCAAATGTTCCCAGA
508	553	0.82	TGAGATGGTTAACTCAAAAGAACGGGAATTATCCTGTTCAAGACGCCCAA
716	761	0.81	GCCCCTTGTCAATGGTCTGATTGGGAGAATTAATTATTATTGGTCTGTAC
777	822	0.91	CAGACATTGCGAGTAAGATCCAATGGGAATCTAATTGCTCCATGGTTCGG
866	911	0.99	AACTGATTTAAACAGTGGCAATTGTGTAGTGCAATGTCAGACTGAAAAAG
900	945	0.9	TGTCAGACTGAAAAAGGTGGCCTAAACAGTACATTACCTTTCCACAATAT
1081	1126	0.97	GAGGTTGGCCAGGGCTAGTTGCCGGTTGGTATGGTTTCCAACATTCAAAC
1174	1219	0.89	AGGCAGTTGACAAAATAACATCCAAGGTGAACAATATAGTCGACAAGATG
1206	1251	0.94	AATATAGTCGACAAGATGAACAAGCAATATGAAATAATTGATCATGAATT
1242	1287	0.88	ATTGATCATGAATTCAGTGAGGTTGAAACTAGACTCAATATGATCAATAA
1251	1296	0.81	GAATTCAGTGAGGTTGAAACTAGACTCAATATGATCAATAACAAAATTGA
1372	1417	0.84	ATGAGCATGACGCAAACGTAAACAACCTATACAACAAAGTGAAAAGGGCC
1404	1449	0.87	AACAAAGTGAAAAGGGCCTTAGGCTCCAATGCAATGGAAGATGGGAAAGG
1486	1531	0.91	AATGCATGGAAACTATTCGGAACGGGACCTATAACAGGAGAAAGTACATG
1572	1617	0.95	GTTAAACTGGAATCTGAGGGGACTTACAAAATACTTACCATTTATTCGAC
1637	1682	0.84	TGTGCTTGCAATGGGGTTTGCTGCCTTCTTATTCTGGGCCATGTCAAATG
1651	1696	0.97	GGTTTGCTGCCTTCTTATTCTGGGCCATGTCAAATGGATCATGCAGGTGC

Table S4. Predicted prokaryote promoter in H9 sequence of H9N2 vaccine strain using Berkeley Drosophila Genome Project (BDGP) program. To decrease E.coli toxic gene expression, prokaryote promoter in Egypt H9 sequence was predicted using BDGP program (https://www.fruitfly.org/seq_tools/promoter.html). The promoter between 1242 to 2387 was adjacent to the repeated nucleotide deletion site and included Shine-Dalgarno sequence (GAGG) and start codon (ATG). The Shine-Dalgarno sequence was mutated into GAAG in this study and cloning of complete H9 genome was succeed.

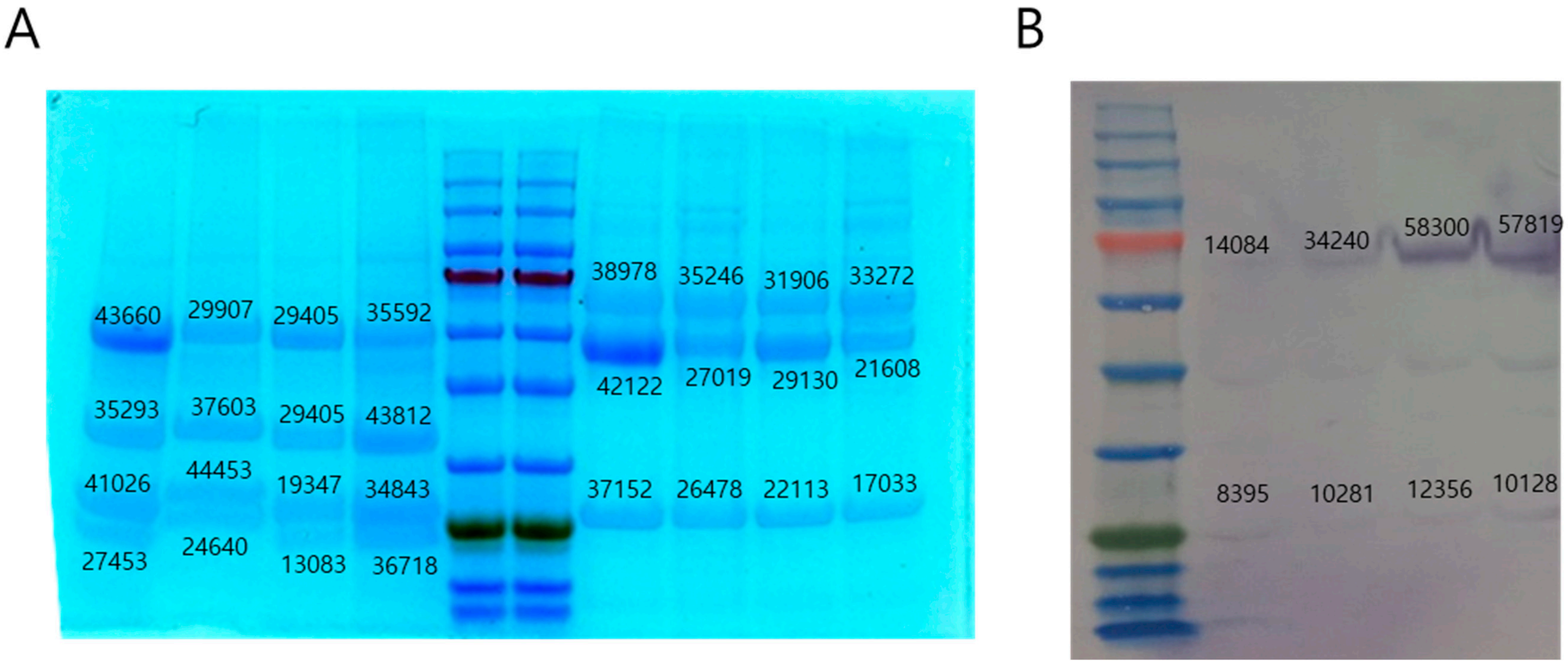

Figure S1. Original unedited figure 9 (A) and 9 (B). Intensity of each bands were measured using ImageJ (https://imagej.nih.gov/ij/download.html) program and the results were indicated near each bands.
